# Barriers and Facilitators to Accessing Care and Adhering to Medications in People With Epilepsy in India: Healthcare Workers’ Perspectives

**DOI:** 10.7759/cureus.72393

**Published:** 2024-10-25

**Authors:** Jack Tomlins, Stephen Pearson

**Affiliations:** 1 Critical Care, King's College Hospital National Health Service (NHS) Foundation Trust, London, GBR; 2 Academic Centre for International Students, Faculty of Arts and Humanities, University of Southampton, Southampton, GBR

**Keywords:** accessing care, adherence to medication, barriers and facilitators, epilepsy, healthcare workers' perspectives, india, qualitative research

## Abstract

Introduction

Exploring the barriers and facilitators that people with epilepsy face in accessing care and adhering to medications could enable a better understanding of how India’s epilepsy treatment gap could be addressed. Furthermore, there is a paucity of research on the topic, and we could not find any studies exploring these barriers from the perspective of healthcare workers.

Aim

The study aimed to explore the barriers and facilitators to accessing care and adhering to medications faced by people with epilepsy in India.

Methodology

Purposive sampling was used to recruit healthcare workers at a private hospital and a non-governmental organization in Pune, India. A total of 13 participants were interviewed, with all of these interviews being audio-recorded. Findings were transcribed and then analyzed by thematic analysis.

Findings

Several barriers to accessing care were identified, with misconceptions surrounding epilepsy being the most frequently mentioned barrier. Facilitators to accessing care mentioned by participants included higher symptom severity and a higher level of education. Several barriers to medication adherence were discussed, with misconceptions and finances being key themes in participants’ responses. Finally, four key themes arose from exploring facilitators to adherence, namely the low cost of medicines, counselling, a good doctor-patient relationship, and a higher education level.

Discussion

The barriers and facilitators in this study were similar to the barriers and facilitators identified in similar studies. However, some key differences were seen too. For example, this study found financial difficulties to be a key barrier to adherence, but a similar study in South India did not find financial difficulties to be a barrier. Several recommendations can be made based on the findings of this study on how to address India’s epilepsy treatment gap.

Conclusion

People with epilepsy in India face several barriers and facilitators to accessing care and adhering to medications. India’s epilepsy treatment gap is a complex and multifactorial issue and will therefore be challenging to address.

## Introduction

Epilepsy is a chronic neurological disorder characterized by recurrent seizures [1]. Seizures are sudden, brief changes to electrical activity in the brain, which can cause, among other symptoms, involuntary movements and loss of consciousness [1]. It is estimated that epilepsy affects around 50 million people globally [1]. Located in South Asia, India has a population of over 1.4 billion [2]. Estimates of epilepsy prevalence in India range from 5.59-10 per 1000 people [3]. Furthermore, this prevalence is rising [4]. The main treatment for epilepsy is the use of medications, known as anti-epileptic drugs (AEDs), which are effective in around 70% of patients [5]. Not taking medications is associated with poorer clinical outcomes in people with epilepsy (PWE) [6]. For example, Al-Aqeel et al. found that PWE who do not take medications have 21% more seizures than those who do take medications [7]. The treatment gap is the percentage of PWE, including those without a diagnosis, who are not receiving treatment [8]. Estimates of India’s epilepsy treatment gap vary but some estimates are as high as 95% [9]. For a patient to be treated effectively with epilepsy medications, two key processes must occur [10]. Firstly, a person with symptoms of epilepsy must access healthcare services to obtain a diagnosis and, if appropriate, obtain a prescription for AEDs [10]. Secondly, they must take and continue to take the medications in line with their doctor’s advice [10], which is known as medication adherence [11]. Therefore, to identify reasons for this treatment gap, one must understand the barriers and facilitators that determine whether PWE access care and adhere to medications. Barriers are factors that make people less likely to access care or adhere to medications, whereas facilitators are factors that make people more likely to engage in these behaviors [12]. Exploring the reasons for India’s epilepsy treatment gap could provide valuable insight into how this issue could be addressed. Despite numerous studies describing that the epilepsy treatment gap is an issue in India, there is a paucity of research investigating the reasons for this treatment gap. Furthermore, we found no studies investigating healthcare workers’ (HWs) perspectives on this treatment gap. Seeking HWs’ perspectives is worthwhile as they are able to offer unique perspectives on barriers and facilitators to treatment, particularly with regard to service issues such as the cost of consultations [13].

Aim

The aim of this study was to identify and explore the barriers and facilitators to accessing care and adhering to medications faced by PWE in India, by exploring HWs’ perspectives on these factors. These findings can then be used to make recommendations about how to increase the proportion of PWE in India who access care and adhere to medications, thereby reducing the epilepsy treatment gap.

## Materials and methods

The research took place in Pune, Western India. Participants were recruited across two sites: Deenanath Mangeshkar Hospital, a private hospital with a neurology department providing treatment for epilepsy, and the Sanvedana Foundation, a non-governmental organization providing support services to PWE, such as counselling and support groups. Purposive sampling was used as it allows the selection of information-rich cases, which are participants who have lots of information to offer on the research topic [14]. A gatekeeper, who worked at both of the research sites, was used to identify HWs who may be appropriate participants in the study. Staff within the two research sites who may have been suitable participants were invited to participate in the study by email. The inclusion criterion was being a HW who works directly with people with epilepsy. Participants who did not speak English were excluded as the interviews were conducted in English. The demographics of the participants are shown in Table 1.

**Table 1:** Participant demographics PWE: People with epilepsy; M: Male; F: Female

Participant code	Occupation	Gender	Years working with PWE
IW1	Technician	M	4
IW2	Technician	F	2
IW3	Medical neuroscientist	M	6
IW4	Neurosurgeon	M	25
IW5	Counsellor	F	22
IW6	Neurologist	M	14
IW7	Neurologist	M	32
IW8	Neurologist	M	8
IW9	Neurologist	M	30
IW10	Counsellor	F	5
IW11	Counsellor	F	8
IW12	Psychiatrist	M	17
IW13	Neurologist	M	30

For this study, a qualitative approach was adopted using a cross-sectional design. The use of qualitative methods enabled the collection of detailed data regarding barriers and facilitators to care access and medication adherence. A cross-sectional design was appropriate as it allows the collection of data at a single point in time, enabling participants to provide information in a descriptive manner [15]. This suited our objectives as we planned to obtain HWs’ knowledge and opinions. Furthermore, a longitudinal design was infeasible as we only had around one month for the data collection phase, and a case study would have been less useful as we would have only obtained one HW’s perspective. Semi-structured interviews were used to elicit findings about barriers and facilitators to care and treatment, as they allow flexibility, enabling participants to offer ideas that may be beyond the interview questions but which are still relevant to the study's objectives [16]. The interview structure was developed using similar studies found through a literature search. The question guide used is shown in the Appendices. In total, 13 interviews were conducted, and all took place in the participants’ workspaces, mostly consultation rooms. Interviews lasted between 20 and 50 minutes and were audio-recorded using a mobile phone with the participants’ consent. Non-verbal cues were noted, such as body language, as they can add emphasis and clarification to participants’ opinions, allowing their feelings to be captured more accurately [17]. The host confirmed in advance that all of the participants spoke English, so an interpreter was not necessary. Ethical approval was obtained from the Leeds Institute of Health Sciences Research Ethics Sub-Committee. Participants received an information sheet detailing the research and were given 24 hours before deciding whether to consent to the study.

The audio-recordings were transcribed using the oTranscribe website (MuckRock Foundation, USA). The exact wording of the participants was transcribed, even in the instance of grammatical errors, to ensure the findings captured the voices of the participants as accurately as possible. Qualitative studies often elicit large volumes of data [18]. Because of this, thematic analysis was used as it allows information to be organized into common themes, enabling the data to be summarised logically whilst retaining a high level of detail [18]. Transcripts were read and colour-coded using a-priori codes, which are codes devised prior to analysis [17]. We developed these a-priori codes using the findings of similar studies. Transcripts were then re-read to colour-code any emerging codes. From these codes, themes were identified and transcripts were re-read again to check that the themes were appropriate for the data. This iterative process ensures data analysis is trustworthy [18]. An audit trail was created in Microsoft Excel (Microsoft Corp., Redmond, USA) to keep a chronological record of the codes as they were added. We selected to do this because using an audit trail increases the confirmability of the research, which is the extent to which the findings actually represent the participants’ opinions [19].

## Results

Findings from the interviews are presented in four sections. Within these sections, findings are organized into themes with some supporting quotations, which are presented alongside their relevant participant code. We have included quotations as their use increases the study’s trustworthiness [20].

Barriers to accessing care

Several key themes arose from participants’ responses about barriers to accessing care. These barriers are presented in Table 2. Five of these barriers were mentioned by multiple participants and four of these were mentioned by just one participant.

**Table 2:** Barriers to accessing care *A person’s caste refers to their social position in Indian society based on a system of social stratification [21].

Barrier	Participants’ perspectives
Misconceptions	10 participants mentioned that misconceptions surrounding epilepsy amongst the population were a barrier to accessing care. They explained that misconceptions were common due to poor public knowledge of epilepsy. The most commonly discussed misconception was people believing that their epilepsy symptoms were caused by religious and supernatural phenomena rather than by a medical condition. For example, IW9 explained that “lots of people don’t think their symptoms are epilepsy, they think that it is God’s wrath or an omen, or a punishment for something they’ve done bad in their life.” Participants explained how these misconceptions lead patients to seek help from non-medical healers, instead of accessing care at the hospital. IW10 explained that “people might think the seizures are black magic and so they don’t think the doctor can help them.” IW12 stated that “many will prefer to see faith healers or astrologers instead of doctors.”
Stigma	Some participants discussed stigma as a barrier. These participants described how epilepsy can be seen as a bad or embarrassing disease in Indian society compared to some diseases that are rarely stigmatized, such as heart diseases. They described how this stigma prevents people from seeking care, predominantly because they are worried about people finding out about them having epilepsy, as well as the discrimination they may suffer as a consequence. IW9 explained how “once you say you have seizures people won’t accept you. You will be removed from school, you will lose friends, and you will not be able to get married. So people think it’s better to not tell anyone about the seizures.”
Finances	Three participants discussed the cost of seeing doctors. IW11 explained that “consultations are usually 800-2000 rupees, which is a lot of money for many Indian people.” One participant explained that government hospitals providing free care are available but that these are very busy so it is very difficult to get an appointment, and consequently, most people don’t use these hospitals.
Location	Participants described how living in rural areas or particular cities can make it harder to access medical facilities or receive care from epilepsy specialists, for example, IW3 described how “some cities don’t have any neurologists.” In regard to rural areas, four participants described how people living in these areas were too far from hospitals and travelling to hospitals was very difficult.
Being female	Six participants mentioned being female as a barrier to accessing care. Three of these participants explained that this was because women are less open about their health, which prevents them from discussing their symptoms with a doctor. For example, IW7, a neurologist, stated that “women are less open, they prefer to hide it.” Participants also explained that women often prioritize their family’s health over their own. One participant described how epilepsy is more of a taboo illness for women than men so they are more likely to experience stigma.
Unaware of services	“They don’t know that there are services which can help their epilepsy.” IW1
Old age	“Older people don’t worry about their health, so won’t see the doctor if they start having seizures.” IW11
Low caste*	“People from lower castes can receive discrimination from hospital staff.” IW5
Denial	“People don’t believe they’re ill, because they are scared of dying." IW5

Interestingly, two participants stated that there were no barriers to accessing epilepsy care and that all PWE access medical care when they start having symptoms. IW8 said, “Actually, I don’t think there is anything stopping people from getting epilepsy care.” However, the participants who said this were both working at a private hospital, which may explain why they don’t believe there are any barriers. This is likely because the PWE they meet are accessing care at the hospital, and they are unlikely to meet PWE who are not using hospital services, so they may be unaware that there are PWE in India who are not accessing care.

Facilitators to accessing care

Questions about facilitators to accessing care received fewer and shorter responses than questions relating to barriers. However, it was still possible to identify four key themes from these responses. These facilitators are shown in Table 3.

**Table 3:** Facilitators to accessing care PWE: People with epilepsy

Facilitator	Participants' perspectives
Severe symptoms	Three participants mentioned that having more severe symptoms increases a PWE’s likelihood of accessing care. Two of these participants described more severe symptoms as meaning increased seizure frequency, whereas one explained that they were referring to the incidence of status epilepticus when they mentioned severe symptoms, which is a seizure lasting longer than five minutes [22].
Higher level of education	Being educated to a higher level was frequently mentioned. IW9 believed that “education is the most important thing because educated people know to see the doctor.” Participants explained how people who are educated are more likely to understand that epilepsy has a medical cause, and therefore, are less likely to have misconceptions. As discussed earlier, the majority of participants felt that misconceptions are a key barrier to accessing care.
Word of mouth	Three participants described that hearing positive anecdotes about people who had used epilepsy services can encourage PWE to access care. For example, IW11 described how “talking to others who’ve been through it makes it less scary.” It was explained that these stories could come from family and friends, as well as doctors telling them stories about patients who’d benefited from epilepsy services. However, for a patient to hear positive stories from a doctor, they must first access healthcare services so this may not be a facilitating factor for people who never see a doctor in the first place.
Good social support	A few participants mentioned social support as a facilitating factor. Most of these spoke about support from family. They explained that this makes it more likely for PWE to use services as family members may encourage them to see a doctor, or may even offer to accompany them to their consultation, making visiting the doctor less daunting. One participant stated the importance of friends in a person’s support system, in addition to family.

Barriers to medication adherence

Five key themes arose from participants’ responses about barriers to medication adherence. These barriers are shown in Table 4.

**Table 4:** Barriers to medication adherence PWE: People with epilepsy

Barrier	Participants' perspectives
Misconceptions	Misconceptions were a key theme in participants’ responses about the barriers to medication adherence, in addition to being mentioned as a barrier to accessing care as discussed earlier. One participant, IW1, explained that these misconceptions usually persist even after visiting the doctor. They stated that “even once they’ve been to the doctor and the doctor explained to them why the medicines are important, they still don’t think they will work as they think their seizures are due to black magic or God’s evil.” Consequently, patients often choose to use non-medical treatments instead. For example, IW13 explained that “lots of people use homeopathy treatments.”
Finances	The most frequently discussed theme was cost. Several participants responded that epilepsy medications were very expensive and that the vast majority of people do not have medical insurance. As a result, many PWE in India cannot afford the medicines. Participants reported that some epilepsy medications are available for free in government hospitals, but as discussed earlier it is very difficult to get an appointment. Furthermore, the medicines are often out of stock. One participant explained that these issues were due to underfunding of epilepsy services and medications by the Department of Health. One participant mentioned that some private hospitals offer discounts to poorer patients, but that these discounts are insufficient. IW5 explained that “in some places poorer people can get their medicines for cheaper than everybody else, but it is still not cheap enough for lots of them.” However, two participants did not believe that finances were a barrier, including IW3 who stated that “cost is not a problem for people in Pune.” This could be explained by the fact that these participants worked in a private hospital, and consequently, all the patients they saw were able to afford private healthcare and therefore could probably afford the medicines. IW10 highlighted that PWE are more likely to be unemployed due to the stigma surrounding their condition, explaining that “it is harder for people who have epilepsy to get a job, many people don’t hire them because of their condition.” They further explained that this lack of income strongly contributes to their inability to afford the medicines they need. Additionally, one participant felt that doctors’ prescribing decisions can contribute to patients being unable to afford the medications. Some epilepsy medications are cheaper than others, but doctors are unaware of this and consequently do not always prescribe the cheapest option. For example, IW12 described how “doctors don’t know which medicines are cheapest, and so the patient doesn’t get the cheapest medicine.”
Side effects	Some participants reported that side effects can prevent PWE from continuing treatment. IW2 described that “when they get side effects they stop taking their medicines.” Additionally, fear of experiencing side effects can prevent PWE from taking medicines in the first place. They described how patients often become aware of potential side effects via the internet. Tiredness, nausea, and headaches were side effects commonly mentioned by participants.
Long treatment length	Participants stated that epilepsy medicines usually have to be taken long-term and that this can put people off taking them. IW5 described how “some patients are keen to have the medicines, but when they are told they have to take it for five years they don’t want them.” Participants explained that epilepsy medications can take a long time to have an effect, and many patients stop taking the medicines if they do not experience instant benefits. Furthermore, one participant, IW13, described that some PWE may even blame the medications for their seizures. IW13 described how “they still have seizures whilst taking the medicines, so they think the medicines must be causing the seizures.”
Location	Two participants mentioned location as a barrier, specifically PWE living rurally. One explained that these people are often poorer so are more likely to struggle to afford the medicines. Both said that PWE living rurally are often a large distance from places that dispense the medicines, so they can’t get the medicines in the first place.

Facilitators to medication adherence

Several themes emerged from participants’ responses about facilitators to medication adherence. Four key facilitators were mentioned by multiple participants, and three additional facilitators were mentioned by only one participant. These facilitators are displayed in Table 5.

**Table 5:** Facilitators to medication adherence PWE: People with epilepsy

Facilitator	Participants' perspectives
Low cost of medications	Two participants felt that epilepsy medications were inexpensive and that this helped PWE to adhere to treatment. For example, IW7 felt that “medicines are cheap nowadays.” This contrasts with the several participants who felt that the medications were expensive. As discussed earlier, these two participants both worked in a private hospital and therefore were unlikely to have met patients who could not afford the medicines, which may explain this finding.
Counselling	Counselling was felt to be crucial in facilitating adherence. For example, IW5 stated that “it is very important to give patients counselling when they start to take the medicines.” One participant described how their hospital offers counselling to PWE upon beginning treatment to explain to them the importance of the medicines and the potential side effects that they may experience. However, another participant believed that most patients are not offered counselling. One participant explained that counselling had further benefits such as stress reduction and better mental health. They felt that both of these factors made it more likely for PWE to continue taking their medications.
Good doctor-patient relationship	Several participants raised the importance of PWE having a good relationship with their doctor. They explained that this allows the patient to be more honest with their doctor when discussing their concerns about medications. For example, IW12 explained that “if they get on with the doctor, they will tell them what side effects they have.” They explained how this then allows the doctor to prescribe a different medication, which may be more suited to the patient and therefore increases the likelihood that they continue taking medications.
Higher level of education	A few participants felt that education was important, predominantly because people with higher levels of education are better able to understand the importance of taking the medications. However, one participant disagreed, explaining that educated people still have misconceptions about the cause of epilepsy. IW1 felt that “education makes no difference because educated people still think that medicines don’t work.”
Severe symptoms	“Lots of people with really bad seizures become obsessed with taking medicines.” IW12
Good social support	“Their family’s support can help them keep taking medicines.” IW9
Easy-going personality	“Easy-going people take medicines because they do what the doctor says.” IW12

## Discussion

As aforementioned, there is a paucity of similar research on the topic, so only limited comparisons can be made between this study and other studies. However, the study findings can contribute some fresh insight into how India's epilepsy treatment gap can be addressed. Misconceptions were by far the most frequently discussed barrier to accessing care. Participants reported that misconceptions are very common in India, predominantly due to poor public knowledge of epilepsy. In a similar study conducted in Uganda, Kaddumukasa et al. also identified misconceptions as a key barrier to accessing care in PWE and similarly found that misconceptions usually occur in people with a poor knowledge of epilepsy [23]. Another important barrier identified in the study was being female. This finding was also identified in an Indian study by von Gaudecker et al. [24]. This suggests that there could be gender inequalities in India’s epilepsy treatment gap, which is concerning. However, we could not find studies investigating whether these gender inequalities exist. This study found a higher education level to be a key facilitator to accessing care, and this was because education can reduce misconceptions. However, the presence of misconceptions regarding epilepsy was identified as a barrier in itself, which demonstrates the interlinking nature of the barriers and facilitators PWE face in accessing care. This highlights the complexity of India's epilepsy treatment gap problem.

Financial difficulties emerged as a key barrier to medication adherence, but this was a point of contention in the participants’ responses as a small minority felt that it was not a barrier. A similar study by Das et al. conducted in a South Indian hospital did not find finances to be a barrier [25]. This could be explained by the difference in study locations as average per capita incomes are higher in South India than in any other region [26]. An interesting explanation identified from this study for PWE not being able to afford medications is that doctors do not always prescribe the cheapest medications. However, the study found several other reasons for financial difficulties, such as the challenges PWE face in finding employment, suggesting that it could be a difficult barrier to address. Three facilitators identified in this study were also found in the study by Das et al., including higher education level, social support, and counselling [25], suggesting that these factors are important facilitators of medication adherence. Counselling is crucial in encouraging adherence in PWE, but unfortunately, most patients are not offered counselling.

Study limitations

Purposive sampling relies on the judgment of the researcher or gatekeeper to select participants and is therefore highly susceptible to researcher bias [27]. To elaborate, the gatekeeper used in this study had expressed their own views in relation to the barriers to care and adherence and may have influenced the selection process towards colleagues with similar views. This, coupled with the small sample size used, reduces the sample’s representativeness. Using a qualitative approach meant that the study was unable to reliably measure how frequently an opinion was seen and therefore rare findings were given the same attention as the more frequent findings. A key limitation of the study is the possible influence that the researchers had on the findings, which is known as research bias [28]. For example, certain questions could have been asked with a different tone of voice during the interviews. Whilst we tried to avoid doing this, research biases often arise subconsciously [28], and it is possible that they influenced our findings to some extent. Triangulation involves using multiple methods to collect data and increases the validity of the findings [29]. However, only one method, which was interviews, was used to collect data in this study. This suggests the study’s validity is limited. We devised some a-priori codes before analyzing the transcripts, but using a-priori codes can reduce the number of emerging codes identified during the analysis process [18]. Therefore, there could be biases in the themes presented in the study findings.

Recommendations based on findings

Several recommendations can be made, based on the study’s findings, for how India’s epilepsy treatment gap could be addressed. Firstly, it may be beneficial to develop a public awareness campaign to educate people in India about epilepsy, including its causes and treatment, with the hope of reducing the prevalence of misconceptions about epilepsy in Indian society. This could be a multi-media campaign, including the use of television, billboards, social media, and newspaper adverts to educate the public. Interventions should be developed to educate doctors and other HWs involved in prescribing epilepsy medications about the relative cost of commonly prescribed AEDs, as well as the importance of prescribing cheap medicines to poorer patients. Further research should be conducted to ascertain whether the epilepsy treatment gap is higher in women than in men in India, as this study identified being female as a barrier to accessing care. This is important because if the treatment gap is found to be higher in women, it may encourage policymakers to develop interventions targeted specifically for women, thereby reducing any gender inequalities in the treatment gap, as well as reducing the treatment gap overall. Finally, a policy could be implemented to ensure doctors offer counselling to PWE when they begin taking medications for their epilepsy, to try to increase their likelihood of adhering to medications. This counselling should educate patients about the importance and potential side effects of the medications they are taking and allow them to discuss any concerns they may have about their diagnosis or treatment.

## Conclusions

PWE in India face several barriers and facilitators to accessing care and adhering to medications. India’s epilepsy treatment gap is a complex and multifactorial issue and will therefore be challenging to address. Misconceptions and financial difficulties are key barriers that contribute to this treatment gap, and a higher education level is a key facilitator. However, due to several methodological limitations of this study, we are reluctant to conclude which of the barriers and facilitators faced by PWE in India are the most critical.

## Appendices

Interview question guide

Barriers to accessing care: What services are available to people with epilepsy in your hospital/centre? Are there any barriers that you think prevent people with epilepsy from accessing these services? Why do you think these barriers exist?

Facilitators to accessing care: Are there any things that you think may help people to access these services, or make it more likely that they access services? How do you think these factors help? Why do you think these facilitating factors exist?

Barriers to drug adherence: What medications are available to people with epilepsy in your hospital/centre? Are there any reasons you think that people with epilepsy who do use health services do not take epilepsy medications? Why do you think these exist?

Facilitators to drug adherence: Are there any things that you think make it more likely that people with epilepsy take medications? How do you think these factors help? Why do you think these facilitating factors exist?

To finalise: Is there anything else you would like to add? Can you think of any ways that I could improve this discussion?
